# Post-Fatigue Fracture and Marginal Behavior of Endodontically Treated Teeth: Partial Crown vs. Full Crown vs. Endocrown vs. Fiber-Reinforced Resin Composite

**DOI:** 10.3390/ma14247733

**Published:** 2021-12-15

**Authors:** Roland Frankenberger, Julia Winter, Marie-Christine Dudek, Michael Naumann, Stefanie Amend, Andreas Braun, Norbert Krämer, Matthias J. Roggendorf

**Affiliations:** 1 Medical Center for Dentistry, Department of Operative Dentistry, Endodontics, and Pediatric Dentistry, Campus Marburg, University Medical Center Giessen and Marburg, Georg-Voigt-Str. 3, 35039 Marburg, Germany; julia.winter@staff.uni-marburg.de (J.W.); marie-christine.dudek@staff.uni-marburg.de (M.-C.D.); anbraun@ukaachen.de (A.B.); matthias.roggendorf@staff.uni-marburg.de (M.J.R.); 2 Department of Prosthodontics, Geriatric Dentistry, and Craniomandibular Disorders, Charité-Universitätsmedizin Berlin, Aßmannshauser Str. 4-6, 14197 Berlin, Germany; michael.naumann@charite.de; 3 Medical Center for Dentistry, Department of Pediatric Dentistry, Campus Giessen, University Medical Center Giessen and Marburg, Schlangenzahl 14, 35392 Giessen, Germany; stefanie.amend@dentist.med.uni-giessen.de (S.A.); norbert.kraemer@dentist.med.uni-giessen.de (N.K.); 4 Department of Operative Dentistry, Periodontology and Preventive Dentistry, RWTH Aachen University, Pauwelsstraße 30, 52074 Aachen, Germany

**Keywords:** endodontically treated teeth, cusp stabilization, fiber-reinforced, resin composites, ceramics, lithium disilicate ceramic, zirconia-reinforced silicate ceramic, zirconia, endocrown

## Abstract

Objectives: To investigate in vitro post-fatigue fracture behavior of endodontically treated molars having been differently restored. Methods: A total of 120 extracted human molars were used. A total of 120 specimens in 14 test groups and one control group (*n* = 8) were root canal treated. After postendodontic sealing and build-up (AdheSE Universal, SDR), additional MOD preparations were cut. Postendodontic restorations were: Direct restorations (Tetric EvoCeram BulkFill bonded with AdheSE Universal and EverX Posterior/Essentia bonded with G-Premio Bond; as filling (F) or direct partial crown (DPC) after reducing the cusps 2 mm; indirect adhesive restorations (partial crown/PC vs. full crown/FC): e.max CAD, Celtra Duo, both luted with Variolink Esthetic; indirect zirconia restorations (partial crown/PC vs. full crown/FC), luted with RelyX Unicem 2; indirect non-bonded cast gold restorations (partial crown/PC vs. full crown/FC; Degunorm), luted with Ketac Cem. Before and after 300,000 thermocycles (5/55 °C) and 1.2 Mio. A total of 100 N load cycles, replicas were analyzed under a SEM for marginal quality in enamel and dentin (where applicable) and finally, specimens were loaded until fracture. Results: In direct groups, there was no difference between RC and FRC in fracture strength (*p* > 0.05); however, direct partial crowns showed higher post-fatigue fracture resistance. Regarding marginal quality, intracoronal FRC restorations exhibited more gap-free margins in enamel than RC. In the indirect groups, there was no significant difference between partial and full crowns in any of the adhesively luted ceramic groups regarding post-fatigue fracture resistance. Zirconia partial crowns exhibited significantly lower marginal quality in enamel. Indirect groups performed significantly better than direct groups in fracture resistance. Within the indirect restorations, both cast gold groups and zirconia full crowns exhibited the highest fracture resistance being superior to control teeth. Significances: Within the limits of this in vitro investigation, it can be concluded that any kind of indirect restoration with cusp replacement is suitable for ETT restoration when a certain cavity extension is exceeded. All indirect restorations, i.e., endocrowns, partial crowns, and full crowns showed a promising performance after in vitro fatigue-loading.

## 1. Introduction

Today there is sufficient evidence that vital teeth may be effectively restored even when substantially decayed [1,2,3,4]; however, after root canal treatment the prognosis is considerably worse [5,6,7] because both pre-existing defects and endodontic access cavities significantly weaken the tooth complex [8,9,10]. Consequently, clinical trials involving endodontically treated teeth (ETT) reported worse results than vital teeth [11,12,13,14,15,16]. As main reasons for clinical failure 12% vertical root fractures, 15% cusp fractures, and 40% periodontal issues have been reported [14]. Both adequate preparation and restoration have been permanently in the focus of primarily in vitro research [17,18,19,20]. In addition, beside the previously investigated issues, also endocrowns have been increasingly focused on [21,22,23,24,25] as special treatment option.

Clinical studies are still the preferable test for dental biomaterials, but they are coming with extremely high efforts and potential patient drop out, and always involve the risk that after several years of clinical service the tested materials are not on the market anymore [1,2,3,4]. This is the reason in vitro studies are so important, primarily when fatigue phenomena are involved [6,7,16,19,26,27,28,29]—nevertheless, of course also in vitro studies have limitations.

In a previous investigation, we evaluated the influence of direct vs. indirect and intracoronal (MO/MOD) vs. coverage restorations (partial crowns) [19]. It was clearly shown that partial crowns always gave more stability to ETT compared to inlays, and the same was true for direct resin composite restorations [19]. Although the mentioned publication involved already 264 teeth, there remained some questions unanswered:

1. Is there a difference between partial crowns and full crowns?
2. Have fiber-reinforced resin composites advantages compared to conventional resin composites in terms of stability [30,31,32,33]?
3. What is the status of endocrowns in that context?

Therefore the null hypotheses of this investigation were: There would be a. no difference between partial, full, and endocrowns irrespective of the material and b. no difference between conventional and fiber-reinforced resin composites. It is the key innovation of this paper to combine fracture strength and marginal quality evaluation, and that it is based on a previously published fundament.

## 2. Methods and Materials

A total of 120 intact, caries- and restoration-free human mandibular wisdom teeth with similar size (max. 3 mm difference) and fully developed and non-damaged roots, extracted for therapeutic reasons were used. All patients were required to give informed consent for inclusion of their extracted teeth. The study was conducted in accordance with the Declaration of Helsinki, and the protocol was approved by the local ethics committee (Project identification code 143/09).

Storage of specimens was performed in an aqueous solution of 0.5% chloramine T at 4 °C for up to 30 days as in any previous studies. Specimens were cleaned of plaque and calculus, and light microscopically investigated that they were free of defects (×20 magnification). The sample size was mainly guided by the maximal capacity of the experimental setup, but it was also in line with previous studies [19,26].

The control group involved non-prepared teeth (*n* = 8), in the remaining 112 specimens in 14 groups (*n* = 8) trepanations were cut and root canal preparation was carried out to a working length of −1 mm from the apical foramen using MTwo rotary instruments (VDW, Munich, Germany) up to size 0.04/#40. Working length was established using a C-Pilot file ISO 10 (VDW) until it was visible at the apical foramen and then −1 mm. Root canals were obturated with gutta-percha (VDW) under lateral compaction with AH Plus sealer (Dentsply DeTrey, Konstanz, Germany), and immediately sealed (Endo-Resto-System with Prime&Bond active and SDR, Dentsply).

**Figure 1:**
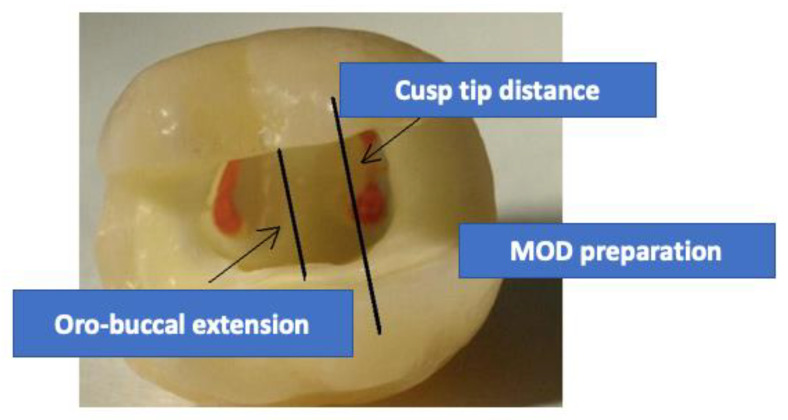
Specimen after MOD preparation and root canal obturation with standardized oro-buccal extension and MOD preparation.

**Figure 2:**
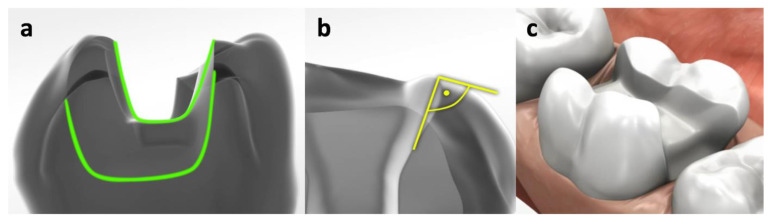
Regimen for fundamental MOD preparations (**a**): rounded angles; (**b**): 90-degree transitions; (**c**): schematic overview. These preps were further prepared to partial or full crowns or filled directly with RC and FRC.

**Figure 3:**
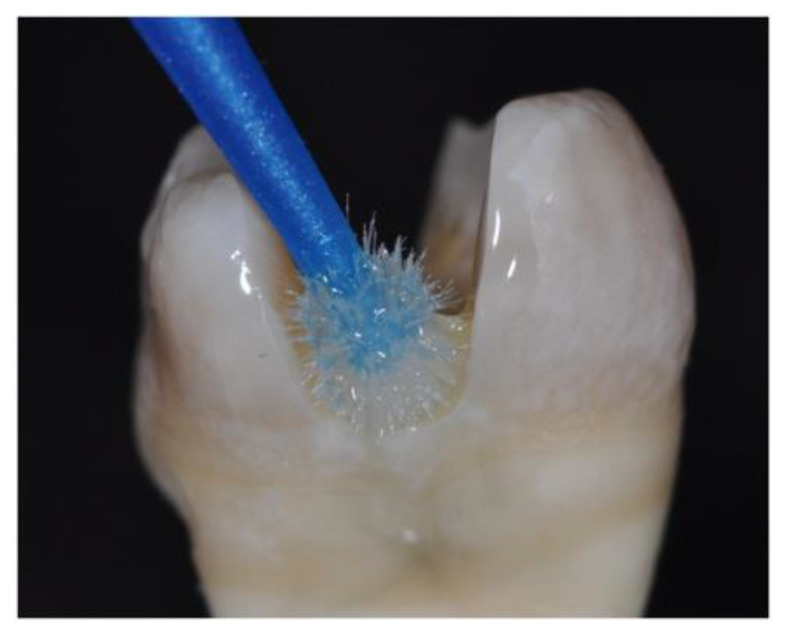
Adhesive pre-treatment in the inlay-style direct resin composite groups.

**Figure 4:**
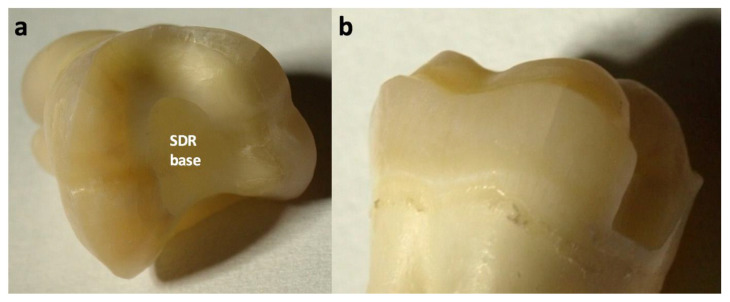
(**a**): Typical ceramic partial crown preparation with central FRC base. (**b**): Classic cast gold preparation with steps and bevels.

Additional to the endodontic access cavities, MOD preparations were cut (Figure 1, Figure 2, Figure 3 and Figure 4). Cavities were prepared with coarse diamond burs under water cooling (80 µm diamond, Komet, Lemgo, Germany/KaVo high-speed handpiece with 3 cooling ports, flow rate 30 mL/min), and finish lines were cut using a 25 µm diamond (one diamond per four cavities). The internal cavity surfaces were cut round, margins did not receive bevels in all indirect preparations. Cavities for direct restorations received a 0.5 mm bevel. All light-cured materials were polymerized using a Bluephase lamp (Ivoclar Vivadent, Schaan, Principality of Liechtenstein). Irradiance was evaluated with a radiometer (Demetron Research Corp, Danbury, CT, USA) to guarantee >1000 mW/cm^2^.

**Figure 5:**
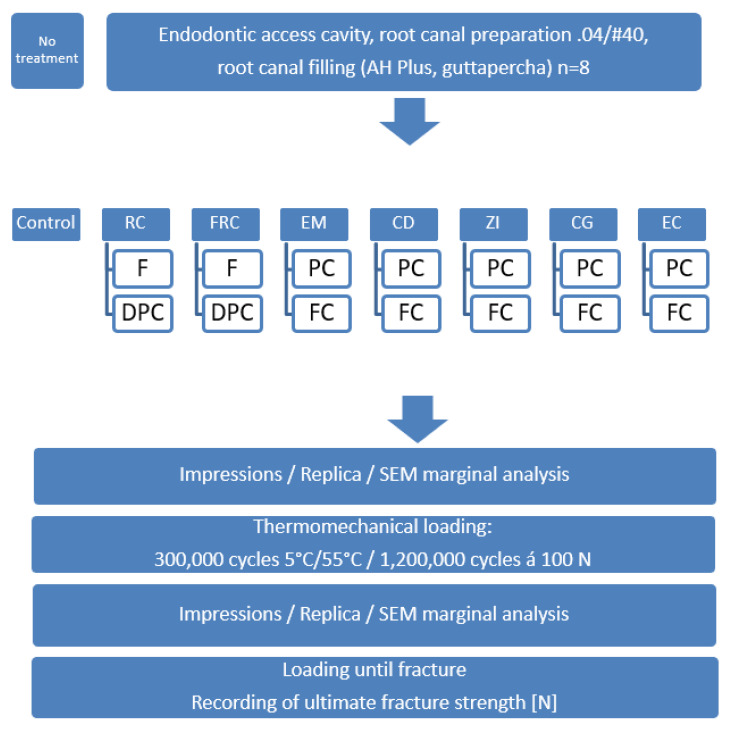
Experimental set up of the study. Abbreviations: F: Filling, DPC: direct partial crown, PC: Partial crown, RC: resin composite, FRC: Fiber-reinforced resin composite, EM: e.max CAD, CD: Celtra Duo, CG: Cast gold, ZI: Zirconia, EC: endocrown.

The involved protocols for restoration are shown in Figure 5. RC procedures: A matrix band was applied to the cavities (MOD) which were bonded with AdheSE Universal (Ivoclar, Figure 3), and restored with Tetric EvoCeram Bulk Fill (Ivoclar) in oblique layers of 2–4 mm thickness. Direct FRC adhesive procedures: After application of a metal matrix, cavities were bonded with G-Premio Bond (GC), and restored with EverX Flow (GC) as dentin substitute being covered with 2 mm Essentia Universal (GC) in same layers as RC. In the DPC groups, all cusps were reduced 2 mm and restorations sculpted coronally. Resin composite layers were polymerized for 40 s each with the curing unit touching the matrix band’s upper edge. Matrices were removed and restorations were additionally polymerized from both sides for 20 s. Marginal overhangs were scaled (A8 S204S, Hu-Friedy, Leimen, Germany) and restorations were polished with flexible disks (SofLex Pop-on, 3M ESPE, St. Paul, MN, USA).

**Figure 6:**
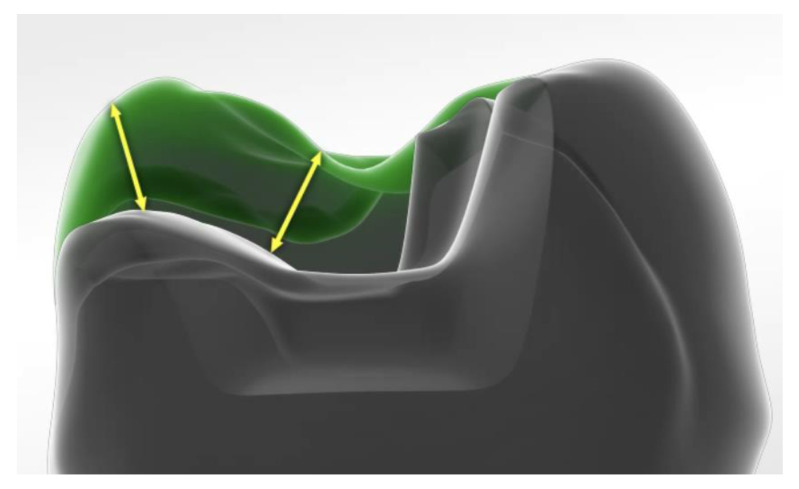
Schematic drawing of replaced cups in the ceramic groups.

Indirect adhesive groups were treated with milled lithium disilicate ceramics (e.max CAD PC/FC, Ivoclar Vivadent, Schaan, Principality of Liechtenstein), zirconia-reinforced lithiumsilicate ceramics (Celtra Duo PC/FC, Dentsply Sirona, Konstanz, Germany), zirconia (Cercon ht, Dentsply Sirona, Konstanz, Germany), and cast gold (Degunorm, Degudent, Hanau, Germany). Endocrowns (PC/FC) were manufactured using e.max CAD. Etchable ceramics were adhesively luted (AdheSE Universal/Variolink Esthetic, Ivoclar Vivadent, Schaan, Principality of Liechtenstein), zirconia was luted with a self-adhesive resin composite cement (RelyX Unicem 2), and cast gold was luted using Ketac Cem (3M Oral Healthcare, Seefeld, Germany). Figure 5 shows the complete methodology, compositions of involved materials are shown in Table 1. CAD/CAM restorations were computed with Cerec 3-D (Sirona, Bensheim, Germany), cast gold was made on traditional dies, zirconia was sintered according to the manufacturer’s recommendations. PC preparations for indirect restorations were carried out as previously described (Figure 4 and Figure 6), cast gold preparations were carried out with step and bevel (Figure 4).

**Figure 7:**
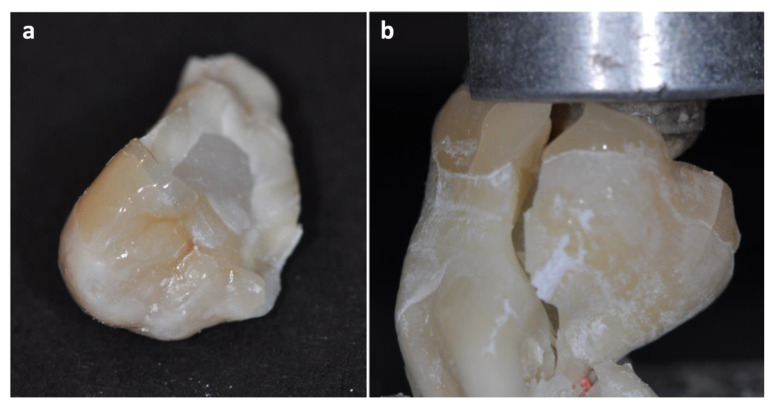
Images of fractured specimens. In every failed restoration, catastrophic fractures were recorded, either vertical (**a**) or oblique (**b**).

Thermomechanical loading of all specimens including controls was carried out in a chewing simulator (CS4 professional line, SD Mechatronik, Feldkirchen, Germany) under water. Liquids such as artificial saliva were not used in order not to falsify marginal quality. Each restored tooth was mounted in one simulator chamber being hit by a steatite antagonist (6 mm diameter, Figure 7b) obliquely chewing on cusps for 1,200,000 cycles at 100 N at a frequency of 0.5 Hz after having been thermocycled 300,000× at +5 °C and +55 °C (THE 1100, SD Mechatronik, Feldkirchen, Germany). The mechanics as well as water temperature within the chewing chambers were periodically reassured for reliable thermomechanical loading (TML). Finally, each specimen was stressed using a universal testing machine (Zwicki, Zwick, Ulm, Germany) with the same antagonist material, the loading cell travelled at 0.5mm/min statically until fracture. Fractured restorations were photographed (Figure 7).

**Figure 8:**
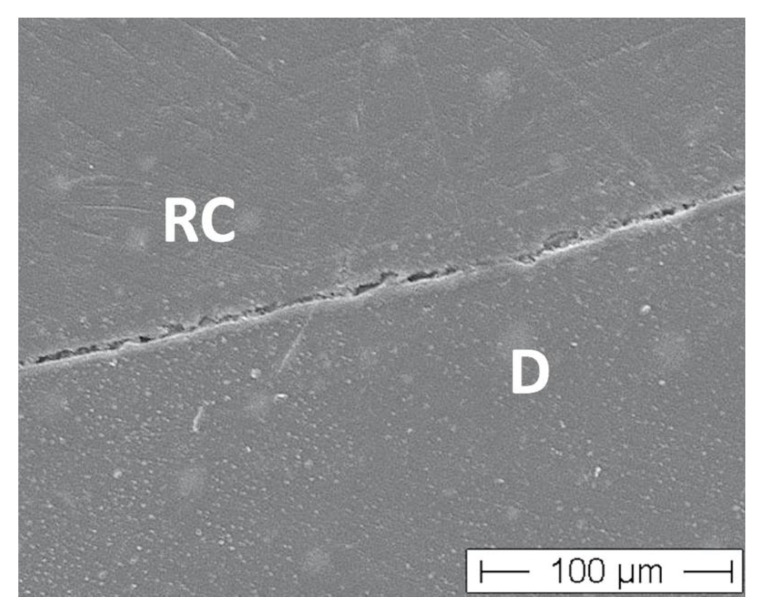
SEM image of marginal gap between dentin (D) and resin composite (RC), 200× magnification.

Both Initially and after completed TML, impressions of the specimens were taken (Provil Novo, Heraeus Kulzer, Hanau, Germany) and replicas (Alpha Die, Schütz Dental, Rosbach, Germany) manufactured. The completed replicas were mounted on aluminum stubs, sputter-coated with gold and examined under a SEM (Phenom, FEI, Amsterdam, The Netherlands) at ×200 magnification. SEM examination was performed by one operator with experience with quantitative margin analysis having been blinded to the restorative procedures. Marginal quality of interfaces (enamel-resin composite, dentin-resin composite, enamel-luting material, dentin-luting material) was expressed as a percentage of the individual margin length in enamel and dentin. Marginal integrity was scored according to the criteria “gap-free margin”, “gap/irregularity” and “not judgeable/artifact” where applicable, i.e., in full crown specimens, no enamel was available (Figure 8). Afterwards the percentage “gap-free margin” in relation to the individual judgeable margin was calculated as marginal quality [19,26], i.e., all visible changes were characterized as “non-gap-free margins”.

To compute statistics, Kolmogorov–Smirnov test was used to show normal distribution of values, so parametric statistical analyses were taken (One-way ANOVA and post hoc Tukey–Kramer test), considering the preparation and restoration techniques as variable. The significance level was set as 5% (SPSS 15.0, SPSS Inc., Chicago, IL, USA).

## 3. Results

The results are displayed in Table 2. In the direct groups, there was no difference between RC and FRC in fracture strength (*p* > 0.05); however, DPC performed significantly better compared to MOD fillings (*p* < 0.05). Regarding marginal quality in enamel, intracoronal FRC restorations exhibited a higher portion of gap-free margins compared to RC restorations (*p* < 0.05). In all other groups, no technique was superior in giving good marginal adaptation after fatigue-loading (*p* > 0.05) with one exception (zirconia partial crowns in enamel with significantly lower scores; *p* < 0.05). Although marginal quality significantly dropped after TML (*p* < 0.05), it remained stable at a very high level (Table 1).

In the indirect groups, there was no significant difference between partial and full crowns in any of the adhesively luted ceramic groups e.max and Celtra Duo regarding post-fatigue fracture resistance (*p* > 0.05). Fully adhesive ceramic restorations gave similar post-fatigue fracture strengths as direct partial crowns of RC and FRC (*p* > 0.05). Groups with cuspal coverage in general performed better than intracoronal restorations (*p* < 0.05), being in different significance levels though, but at a generally high level throughout the groups.

Zirconia FC as well as cast gold PC/FC yielded the highest post-fatigue fracture resistance being even superior to sound teeth of the control group (*p* < 0.05).

## 4. Discussion

As mentioned in the introduction, clinical trials remain the ultimate instrument in restorative dentistry [2,4,11,14]. Main disadvantage of these trials is that some interesting experimental groups may not be accepted by IRBs due to their explorative and less data-supported character. Therefore, it still makes sense in biomaterials research to simulate clinical circumstances to predict clinical behavior [12,16,20,25,27,29]. Additionally, when this is performed meticulously, it remains an in vitro study with several limitations such as standardized loading, lack of sliding bruxism, and more or less rigid fixation during TML.

Regarding the restoration of vital teeth, overall sealing properties, abrasion characteristics, and biological issues such as biodegradation and absence of postoperative hypersensitivities are of primary interest [1,4,26]. With ETT, fracture behavior was regularly investigated because 27% of clinical failures have been linked to any kind of fracture [14,15,27,28]. In this context, fatigue-loading has significantly gained importance for both evaluation of long-term adhesion and fracture resistance [19,26,29]. Clinical recordings showed average masticatory forces around 20 MPa with dramatically higher peak load [5,10,15,29]. At least empirically, a lower threshold level for tactile sensitivity was reported for ETT. Although this was not completely confirmed in the literature, it is common sense that ETT exhibit a significantly higher fracture risk compared to vital teeth [15,28,29]. The main reason for increased fracture risk of ETT is their intentional hard tissue reduction during endodontic access cavity and root canal preparation [6,7,10]. This may be the reason for the observation that full crown preparations are recommended for ETT, also when focusing on clinical outcome of direct restorations of ETT [10,11,12,13].

The methodology of this in vitro study obviously gave reproducible results with different materials, also matching several clinical observations, and having been successfully established since >25 years [19,26]. Long-term thermomechanical fatigue-loading is estimated to be closer to intraoral conditions compared to ultimate loading until fracture [26]. Compared to previous investigations on “vital” teeth, both higher fatigue load and increased number of thermomechanical load cycles was chosen as shown before [19].

The chosen restorative materials were traditional vs. recent biomaterials. The first null hypothesis was that conventional resin composites and fiber-reinforced composites would behave similar, although reports about short-fiber-reinforced composite were favorable [30]. The similarity in in vitro performance, however, correlated well with biomechanical properties of the investigated materials. It could be again shown that partial coverage was more effective in both marginal and fracture behavior in the direct groups (*p* < 0.05). Between the groups RC and FRC, no statistically different results occured in post-fatigue fracture resistance; however, there was a significantly higher portion of gap-free margins in enamel when the fiber-reinforced dentin substitute everX posterior was used as in intracoronal restorations (*p* < 0.05). In all other criteria, there was no beneficial effect of short-fiber-reinforced composite (*p* > 0.05). As in the previous investigation, it could not be confirmed that resin-based materials give less catastrophic failures compared to e.g., ceramics. Altogether the opposite seems to be true, the advantage of direct restorations to be less invasive did not result in superior post-fatigue resistance, because indirect approaches were more effective in general during the present in vitro investigation.

So finally, both null hypotheses had to be accepted because the type of direct material had no impact on fracture strength, and there was no considerable difference between partial crown and full crown preparation in most of the test groups, so the less invasive partial crown can also be recommended for restoration of ETT. Altogether, previous findings could be confirmed that cuspal coverage as well as full crowns perform best with a clear advantage for cast gold restorations as partial or full crown.

## 5. Conclusions

Within the limits of this in vitro investigation, it can be concluded that any kind of indirect restoration with cuspal coverage is suitable for the restoration of ETT when a certain cavity extension is exceeded. All indirect restorations, i.e., endocrowns, partial crowns, and full crowns showed a promising performance after in vitro fatigue-loading.

## Figures and Tables

**Table 1:** Materials under investigation.

Restorative Material	Classification	Composition (%wt)	Manufacturer
Tetric EvoCeram Bulk Fill	Nanohybrid resin composite	Dimethacrylate, prepolymer, Barium glass, Ytterbiumtrifluoride, mixed oxides, initiators, stabilizators	Ivoclar Vivadent, Schaan, Principality of Liechtenstein
everX flow posterior	Fiber-reinforced bulk-fill composite	(1-Methylethyliden) bis [4,1-phenyleneoxy (2-hydroxy-3,1- propanediyl)] bismethacrylate, 2,2′-Ethylenedioxydiethyldimethacrylat, Diphenyl(2,4,6-trimethylbenzoyl) phosphinoxid, 6-T ert-butyl-2,4-xylenol 0.2%, short glass fibers, barium glass	GC Germany, Bad Homburg, Germany
Essentia Universal	Fine hybrid resin composite	7,7,9(or 7,9,9)-trimethyl-4,13-dioxo-3,14-dioxa-5,12-diazahexadecane-1,16-diyl bismethacrylate, Ytterbium trifluoride, (octahydro-4,7-methano-1H-indenediyl) bis (methylene) bismethacrylate, Esterification products of 4,4′-isopropylidenediphenol, ethoxylated and 2- methylprop-2-enoic acid, 2-(2H-benzotriazol-2-yl)-p-cresol, glass fillers	
e.max CAD	Lithium disilicate ceramic	SiO2, Li_2_O, K_2_O, P_2_O_5_, ZrO_2_, ZnO, ZnO, Al_2_O_3_, MgO	Ivoclar Vivadent, Schaan, Principality of Liechtenstein
Celtra Duo	Zirconia-reinforced lithium silicate ceramic	Lithium silicate with 10% ZrO_2_	Dentsply Sirona, Konstanz, Germany
Cercon ht	Zirconia	zirconium oxide, yttrium oxide, hafnium oxide, Aluminum oxide, Silicon oxide	Dentsply Sirona, Konstanz, Germany
Degunorm	Cast gold	73.8% Au, 9% Pt, 9.2% Ag, 4.4% Cu, 2% Zn, 1.5% In, 0.1% Ir	Degudent, Hanau, Germany
Luting Material			
G-Premio Bond	2-step universal adhesive	Etchant: 36% phosphoric acid Universal adhesive: 10-MDP, 4-META, 10-MDTP, methacrylate acid ester, distilled water, acetone, initiators fine powdered silica	GC Germany, Bad Homburg, Germany
AdheSE Universal	2-step universal adhesive	Etchant: 36% phosphoric acid Universal adhesive: 2-hydroxyethyl methacrylate, Bis-GMA, Ethanol, 1,10-decandiol dimethacrylate, methacrylated phosphoric acid ester, campherquinone, 2-dimethylaminoethyl mathacrylate	Ivoclar Vivadent, Schaan, Principality of Liechtenstein
Variolink Esthetic	Luting resin composite	Base: Bis-GMA, UDMA, TEGDMA, fillers, ytterbium trifluoride, stabilizers, pigments Catalyst: Bis-GMA, UDMA, TEGDMA, fillers, ytterbium trifluoride, stabilizers, pigments, benzoyl peroxide	
RelyX Unicem 2	Self-adhesive resin cement	Base: methacrylate monomers with phosphpric acid groups, methacrylate monomers, silanated fillers, initiator components, rheological additives Catalyst: methacrylate monomers, alkaline fillers, silanated fillers, initiator compoments, stabilizers, pigments, rheological additives	3M Oral Healthcare, Seefeld, Germany
Ketac Cem	Luting glass ionomer cement	Powder: calcium FASG Liquid: copolymer of acrylic and maleic acid + water	

**Table 2:** Results [N] ± SD for fracture strength and results [%] (SD) for marginal quality as percentage of “gap-free margins”.

Group	Fracture Strength after TML in N ± SD	Gap-Free Margins Enamel Initial in %(SD)	Gap-Free Margins Enamel after TML in %(SD)	Gap-Free Margins Dentin Initial in %(SD)	Gap-Free Margins Dentin after TML in %(SD)
Control	806 ± 190 ^B^	n/a	n/a	100	n/a
RC-F	382 ± 83 ^D^	100	82 (13) ^B^	100	n/a
RC-DPC	688 ± 186 ^C^	100	88 (9) ^A^	100	n/a
FRC-F	402 ± 110 ^D^	100	89 (10) ^A^	100	n/a
FRC-DPC	699 ± 178 ^C^	100	93 (9) ^A^	100	n/a
EM-PC	723 ± 188 ^C^	100	95 (7) ^A^	100	n/a
EM-FC	736 ± 160 ^C^	n/a	n/a	100	95 (5) ^A^
CD-PC	702 ± 167 ^C^	100	93 (9) ^A^	100	n/a
CD-FC	733 ± 152 ^C^	n/a	n/a	100	96 (4) ^A^
ZI-PC	702 ± 143 ^C^	100	76 (23) ^C^*	100	n/a
ZI-FC	921 ± 102 ^A^	n/a	n/a	100	94 (8) ^A^
CG-PC	934 ± 172 ^A^	100	90 (5) ^A^	100	n/a
CG-FC	956 ± 200 ^A^	n/a	n/a	100	93 (5) ^A^
EC-PC	689 ± 175 ^C^	100	88 (12) ^A^	100	n/a
EC-FC	734 ± 197 ^C^	n/a	n/a	100	94 (5) ^A^

*: compared to the other groups under investigation, in this group marginal irregularities were predominantly recorded between enamel and luting composite. Superscript letters: Same letters mean *p* > 0.05 within columns. Before and after TML, a significant decrease in marginal quality was recorded in all groups (*p* < 0.05).

## Data Availability

The data presented in this study are available on request from the corresponding author.
